# Supplementary material for: A Comparative Study of Microbial Communities, Biogenic Amines, and Volatile Profiles in the Brewing Process of Rice Wines with Hongqu and Xiaoqu as Fermentation Starters
Source: Foods. 2024 Aug 2;13(15):2452. doi: 10.3390/foods13152452 (PMC11311568; doi:10.3390/foods13152452)
Supplement: Supplementary file 1 [file foods-13-02452-s001.zip › foods-3001236-supplementary.pdf]

Supplementary materials

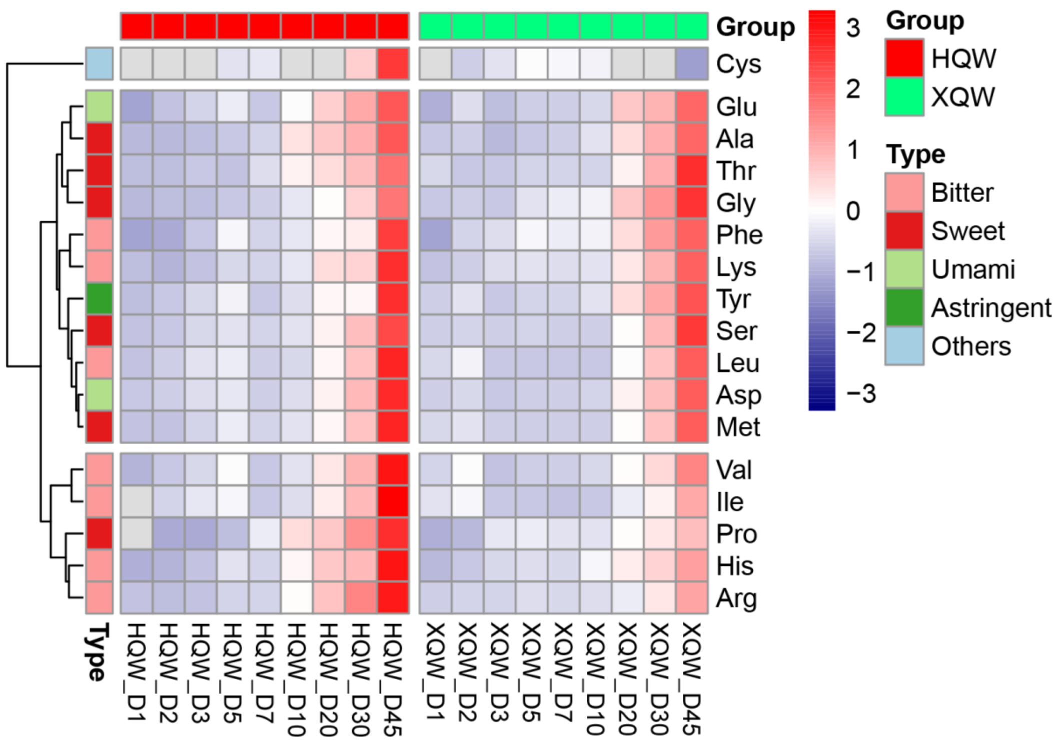

Figure S1 The dynamics of amino acid content during HQW and XQW brewing.

Table S1. Microbial contribution to the enzymes for the metabolism of volatile profiles during Hongqu rice wine and Xiaoqu rice wine brewing

Pathway	Enzyme Name	Enzyme No.	XQW Distribution of microbes	XQW EC abundance	HQW Distribution of microbes	HQW EC abundance
Glycolysis	Glycogen phosphorylase	2.4.1.1	*Weissella confusa* (32.06%), *Pantoea dispersa* (22.02%), *Pantoea*_sp._AG1095 (11.86%), *Kosakonia cowanii* (5.94%), *Cronobacter sakazakii* (3.12%), unclassified_g_*Enterobacter* (3.09%), unclassified_o_*Enterobacterales* (3.04%)	52424.91	*Weissella cibaria* (55.53%), *Pantoea dispersa* (15.46%), *Pantoea* AG1095 (9.91%), unclassified_o_*Enterobacterales* (4.31%), unclassified_f_*Enterobacteriaceae* (2.61%), *Kosakonia cowanii* (2.56%), *Klebsiella pneumoniae* (2.14%)	48925.78
	Glucan 1, 4-alpha-glucosidase	3.2.1.3	*Saccharomycopsis fibuligera* (64.98%), *Rhizopus delemar* (23.86%), *Rhizopus microsporus* (5.13%), *Saccharomyces cerevisiae* (2.51%), *Rhizopus oryzae* (2.47%)	2607.92	*Saccharomyces cerevisiae* (33.25%), *Pantoea ananatis* (24.74%), *Monascus purpureus* (24.61%), *Pantoea*_sp._OXWO6B1 (5.98%), *Rhizopus oryzae* (5.76%), *Byssochlamys spectabilis* (5.67%)	327.26
	Amylo-alpha-1, 6-glucosidase	3.2.1.33	*Ascoidea rubescens* (49.37%), *Rhizopus delemar* (25.36%), *Lichtheimia ramosa* (11.63%), *Saccharomyces cerevisiae* (10.09%), *Lachancea fermentati* (2.56%)	1693.69	*Saccharomyces cerevisiae* (86.15%), *Monascus purpureus* (13.85%)	632.53
	Glucose-1-phosphatase	3.1.3.10	Unclassified_g_ *Pantoea* (53.78%), *Enterobacter cloacae* (16.04%), *Enterobacter roggenkampii* (5.68%), *Kosakonia cowanii* (5.48%), unclassified_g_*Cronobacter* (3.78%), *Enterobacter kobei* (2.77%), *Lactococcus lactis* (2.5%)	16865.32	unclassified_g_*Pantoea* (65.39%), *Klebsiella pneumoniae* (8.34%), *Enterobacter cloacae* (4.5%), *Klebsiella*_cf._*planticola* B43 (3.79%), *Kosakonia cowanii* (3.49%), unclassified_f_*Enterobacteriaceae* (2.78%)	9934.81

Alpha-amylase	3.2.1.1	*Enterobacter asburiae* (14.27%), *Enterobacter roggenkampii* (13.75%), *Kosakonia cowanii* (13.11%), *Cronobacter dublinensis* (10.86%), *Enterobacter bugandensis* (9.8%), unclassified_g_*Enterobacter* (6.6%)	15906.42	*Burkholderia gladioli* (52.34%), *Enterobacter asburiae* (7.83%), *Klebsiella pneumoniae* (6.45%), unclassified_f_*Enterobacteriaceae* (6.38%), unclassified_g_*Enterobacter* (4.87%), *Kosakonia cowanii* (4.81%), *Enterobacter bugandensis* (4.47%)	19656.59
Maltogenic alpha-amylase	3.2.1.133	*Weissella confusa* (92.43%), *Leuconostoc lactis* (4.12%), *Leuconostoc*_sp. (2.3%)	10998.29	*Leuconostoc lactis* (88.35%), *Lactococcus garvieae* (9.54%), *Weissella confusa* (1.72%)	836.16
Alpha-glucosidase	3.2.1.20	*Weissella cibaria* (39.12%), *Pantoea*_sp._Ap-870 (10.71%), *Enterobacter asburiae* (9.33%), *Cronobacter dublinensis* (8.06%), *Pediococcus pentosaceus* (6.46%), *Cronobacter sakazakii* (4.51%), *Kosakonia*_sp._CCTCC M2018092 (3.36%)	31242.36	*Weissella cibaria* (64.9%), *Pantoea*_sp._Ap-870 (10.93%), *Enterobacter asburiae* (6.04%), unclassified_f_*Enterobacteriaceae* (3.57%), unclassified_g_*Klebsiella* (3.28%)	31044.97
4-Alpha-glucanotransferase	2.4.1.25	*Pantoea dispersa* (23.7%), *Pantoea*_sp._BK028 (21.31%), *Cronobacter dublinensis* (7.08%), *Kosakonia cowanii* (6.62%), *Enterobacter* cloacae (6.4%), *Enterobacter hormaechei* (6.17%), *Cronobacter sakazakii* (4.39%), *Ascoidea rubescens* (4.13%)	20251.95	*Pantoea dispersa* (33.52%), *Pantoea*_sp._BK028 (23.09%), unclassified_f_*Enterobacteriaceae* (10.23%), *Kosakonia cowanii* (4.68%), *Enterobacter hormaechei* (4.51%), *Saccharomyces cerevisiae* (4.5%)	12105.80
Maltose phosphorylase	2.4.1.8	*Pediococcus pentosaceus* (56.43%), *Weissella confusa* (31.76%), unclassified_g_*Lactobacillus* (6.7%), *Leuconostoc mesenteroides* (2.31%), *Leuconostoc lactis* (1.24%), *Weissella cibaria* (1.19%)	37340.49	*Weissella cibaria* (88.14%), *Leuconostoc mesenteroides* (6.58%), *Leuconostoc lactis* (4.7%)	18848.45

Oligo-1, 6-glucosidase	3.2.1.10	*Pediococcus* pentosaceus (58.24%), *Saccharomyces cerevisiae* (21.27%), unclassified_g_*Cronobacter* (12.49%), *Weissella paramesenteroides* (1.99%), *Leuconostoc pseudomesenteroides* (1%)	6693.94	*Leuconostoc citreum* (44.98%), *Saccharomyces cerevisiae* (28.16%), *Leuconostoc lactis* (14.3%), *Pediococcus damnosus* (3.52%), *Franconibacter pulveris* (2.55%), *Monascus purpureus* (2.36%)	3396.96
Phosphoglucomutase	5.4.2.2	*Pediococcus pentosaceus* (58.06%), unclassified_g_*Pantoea* (13.52%), unclassified_g_*Weissella* (5.86%), *Lelliottia nimipressuralis* (5.47%), *Weissella confusa* (4.2%)	41753.55	unclassified_g_*Weissella* (50.97%), unclassified_g_*Burkholderia* (24.12%), unclassified_g_*Pantoea* (12.53%), *Lelliottia nimipressuralis* (3.39%)	32754.05
Hexokinase	2.7.1.1	*Rhizopus delemar* (39.69%), *Ascoidea rubescens* (18.12%), *Hyphopichia burtonii* (14.19%), *Saccharomyces cerevisiae* (12.02%), *Babjeviella* inositovora (10.35%), *Rhizopus microsporus* (3.28%)	4550.03	*Saccharomyces cerevisiae* (81.15%), *Monascus purpureus* (18.65%)	799.59
Glucokinase	2.7.1.2	*Pediococcus pentosaceus* (67.08%), unclassified_g_*Pantoea* (13.12%), unclassified_g_*Weissella* (11.36%), *Enterobacter asburiae* (1.95%), unclassified_g_Cronobacter (1.78%), unclassified_f_*Enterobacteriaceae* (1.53%)	33620.72	unclassified_g_*Weissella* (43.43%), unclassified_g_*Burkholderia* (24.71%), *Burkholderia gladioli* (15.61%), unclassified_g_*Pantoea* (10.49%), unclassified_f_*Enterobacteriaceae* (2.93%)	35893.81
Polyphosphate glucokinase	2.7.1.63	*Burkholderia gladioli* (48.88%), *Pantoea ananatis* (24.77%), unclassified_g_*Rhodococcus*_f_*Nocardiaceae* (20.22%), *Saccharopolyspora hirsuta* (6.13%)	28.29	*Burkholderia gladioli* (98.87%), *Pantoea ananatis* (1.13%)	78.82
Glucose-6-phosphate isomerase	5.3.1.9	unclassified_g_*Pediococcus* (44.24%), *Weissella cibaria* (25.48%), unclassified_g_*Pantoea* (10.23%)	54195.48	*Weissella cibaria* (52.22%), *Burkholderia gladioli* (22.76%), unclassified_g_*Pantoea* (10.99%), *Klebsiella pneumoniae* (4.26%), *Enterobacter hormaechei* (1.63%)	36713.50

6-phosphofructokinase	2.7.1.11	unclassified_g_*Pediococcus* (56.23%), *Rhizopus microsporus* (21.46%), unclassified_f_*Enterobacteriaceae* (4.9%), *Wickerhamomyces* anomalus (4.72%), unclassified_g_*Enterobacter* (2.14%), *Enterobacter roggenkampii* (1.89%), *Cronobacter dublinensis* (1.59%)	8549.82	*Klebsiella pneumoniae* (40.24%), unclassified_f_*Enterobacteriaceae* (23.03%), *Saccharomyces cerevisiae* (7.94%), unclassified_g_*Enterobacter* (7.52%), *Enterobacter*_sp._UCD-UG FMILLET (5.87%), unclassified_g_*Pantoea* (2.5%)	4406.25
diphosphate-dependent phosphofructokinase	2.7.1.90	*Rhodococcus* erythropolis (100%)	3.47	*Rhodococcus erythropolis* (50%), unclassified_g_*Variovorax* (50%)	1.68
Fructose-bisphosphate aldolase, class II	4.1.2.13	unclassified_g_*Pediococcus* (40.42%), unclassified_g_*Pantoea* (27.47%), *Weissella* confusa (22.74%), unclassified_o_*Enterobacterales* (10.29%), *Pantoea dispersa* (9.72%), *Pediococcus pentosaceus* (8.47%), unclassified_g_*Enterobacter* (6.16%), unclassified_f_*Enterobacteriaceae* (4.55%)	48900.98	unclassified_g_*Pantoea* (33.76%), unclassified_g_*Burkholderia* (25.8%), *Pantoea dispersa* (12.11%), unclassified_o_*Enterobacterales* (8.39%), unclassified_f_*Enterobacteriaceae* (4.14%), *Klebsiella pneumoniae* (3.66%)	21591.46
Triosephosphate isomerase (TIM)	5.3.1.1	*Pediococcus pentosaceus* (48.09%), *Weissella confusa* (37.75%), *Weissella cibaria* (2.66%), unclassified_f_*Enterobacteriaceae* (2.45%), unclassified_g_*Pantoea* (1.69%), *Ascoidea rubescens* (1.52%), *Enterobacter*_sp._OV724 (1.15%)	41954.72	*Weissella cibaria* (70.54%), *Burkholderia gladioli* (18.57%), unclassified_f_*Enterobacteriaceae* (3.39%)	38584.46
Glyceraldehyde 3-phosphate dehydrogenase	1.2.1.12	unclassified_g_*Pediococcus* (55.1%), Weissella *cibaria* (14.22%), unclassified_g_*Enterobacter* (3.69%), *Pantoea*_sp._BK028 (2.97%), unclassified_o_*Enterobacterales* (2.8%), *Enterobacter cloacae* (2.72%)	42339.64	*Weissella cibaria* (50.78%), *Burkholderia gladioli* (25.96%), *Pantoea*_sp._BK028 (4.98%), *Leuconostoc citreum* (2.64%)	32780.68

Phosphoglycerate kinase	2.7.2.3	unclassified_g_*Pediococcus* (70.45%), *Weissella cibaria* (12.07%), unclassified_g_*Pantoea* (5.3%), unclassified_g_*Cronobacter* (3.85%), *Debaryomyces fabryi* (2.13%)	33037.74	*Weissella cibaria* (53.52%), unclassified_g_*Burkholderia* (32.17%), unclassified_g_*Pantoea* (4.36%), *Leuconostoc lactis* (2.99%)	27760.84
2, 3-Bisphosphoglycerate-independent phosphoglycerate mutase	5.4.2.12	*Kosakonia cowanii* (25.04%), unclassified_g_*Cronobacter* (19.69%), *Kosakonia oryziphila* (10.2%), *Enterobacter* roggenkampii (9.66%), *Cronobacter malonaticus* (8.03%)	4140.80	*Kosakonia cowanii* (30.9%), *Klebsiella pneumoniae* (29.23%), *Kosakonia oryziphila* (6.28%), *Cronobacter malonaticus* (6.1%), *Enterobacter ludwigii* (6.08%), unclassified_g_*Enterobacter* (4.96%), *Franconibacter pulveris* (4.6%)	2628.56
glycerate phosphomutase	5.4.2.11	unclassified_g_Pediococcus (30.77%), *Pediococcus pentosaceus* (28.97%), unclassified_g_Pantoea (6.46%), *Pantoea*_sp._R102 (6.21%), unclassified_g_Weissella (6.09%), unclassified_f_*Enterobacteriaceae* (5.99%), *Pantoea ananatis* (5.34%)	65859.90	unclassified_g_*Weissella* (34.96%), *Burkholderia gladioli* (32.64%), unclassified_g_*Pantoea* (7.97%), *Pantoea*_sp._R102 (7.78%), unclassified_f_*Enterobacteriaceae* (6.23%)	42180.07
Enolase	4.2.1.11	unclassified_g_*Pediococcus* (43.62%), *Pediococcus pentosaceus* (20.2%), *Weissella cibaria* (13.37%), unclassified_f_*Enterobacteriaceae* (8.65%), unclassified_g_*Pantoea* (5.7%), *Komagataella phaffii* (1.68%)	54612.52	*Weissella cibaria* (51.43%), unclassified_g_*Burkholderia* (28.23%), unclassified_f_*Enterobacteriaceae* (11.89%), unclassified_g_*Leuconostoc* (3.09%)	30913.55
Pyruvate kinase	2.7.1.40	unclassified_g_*Pediococcus* (51%), *Weissella cibaria* (13.07%), unclassified_g_*Pantoea* (10.78%), *Cronobacter malonaticus* (3.53%), *Enterobacter*_sp._*Crenshaw* (2.77%), *Hyphopichia burtonii* (2.2%),	39156.41	*Weissella cibaria* (39.92%), *Burkholderia gladioli* (38.95%), unclassified_g_*Pantoea* (7.67%)	17249.01
pyruvate, orthophosphate dikinase	2.7.9.1	*Staphylococcus gallinarum* (100%)	16.55	*Lactococcus garvieae* (95.33%), *Bacillus ginsengihumi* (4.67%)	62.60

	pyruvate, water dikinase	2.7.9.2	unclassified_g_*Pantoea* (53%), *Kosakonia* cowanii (10.29%), *Lelliottia nimipressuralis* (8.37%), unclassified_f_*Enterobacteriaceae* (7.92%), *Enterobacter cloacae* (7.51%), *Cronobacter malonaticus* (4.11%), *Enterobacter*_sp._638 (3.62%)	9512.77	unclassified_g_*Burkholderia* (59.72%), unclassified_g_*Pantoea* (25.15%), unclassified_f_*Enterobacteriaceae* (6.19%)	15111.00
	Acetolactate synthase	2.2.1.6	unclassified_g_*Pediococcus* (29.39%), unclassified_g_*Pantoea* (21.93%), *Pediococcus pentosaceus* (9.58%), unclassified_g_*Enterobacter* (5.54%), *Kosakonia cowanii* (4.1%), *Enterobacter cloacae* (3.68%), *Weissella cibaria* (3.41%), unclassified_f_*Enterobacteriaceae* (2.89%)	87080.72	unclassified_g_*Burkholderia* (23.39%), *Weissella cibaria* (21.11%), *Burkholderia gladioli* (20.33%), unclassified_g_*Pantoea* (15.35%), unclassified_f_*Enterobacteriaceae* (3.73%)	87086.38
Higher alcohols biosynthesis	Ketol-acid reductoisomerase (NADP+)	1.1.1.86	*Kosakonia cowanii* (26.74%), *Pantoea*_sp._ARC607 (12.28%), *Enterobacter hormaechei* (12.03%), unclassified_g_*Cronobacter* (10.89%), *Candida haemuloni* (7.75%), *Pantoea ananatis* (6.37%), *Pantoea agglomerans* (5.89%), *Klebsiella pneumoniae* (5.35%)	9156.39	unclassified_g_*Burkholderia* (65.63%), *Kosakonia cowanii* (10.82%), *Enterobacter hormaechei* (5.77%), *Pantoea*_sp._ARC607 (4.59%)	12222.15
	Dihydroxy-acid dehydratase	4.2.1.9	Pantoea_sp._R102 (28%), *Enterobacter cloacae* (18.38%), *Salmonella enterica* (7.23%), unclassified_d_*Bacteria* (4.98%), *Hanseniaspora* osmophila (4.83%), *Kwoniella mangrovensis* (4.72%), *Escherichia* coli (4.63%), *Pantoea ananatis* (3.84%)	16159.34	*Burkholderia gladioli* (51.92%), *Burkholderia*_sp._SJZ089 (23.81%), *Pantoea*_sp._R102 (9.23%), unclassified_d_*Bacteria* (4.23%)	34389.18
	Valine dehydrogenase (NAD+)	1.4.1.23	*Rhodococcus* qingshengii (100%)	1.73	*Rhodococcus qingshengii* (100%)	5.00

Valine-pyruvate aminotransferase	2.6.1.66	*Enterobacter ludwigii* (37.66%), *Pantoea dispersa* (24.83%), unclassified_f_*Enterobacteriaceae* (15.96%), *Enterobacter asburiae* (6.19%), *Cronobacter malonaticus* (3.4%), *Cronobacter sakazakii* (2.79%), *Enterobacter*_sp._Acro-832 (1.78%), *Kosakonia cowanii* (1.48%)	19207.34	*Burkholderia gladioli* (31.91%), *Enterobacter ludwigii* (23.71%), *Pantoea dispersa* (16.96%), unclassified_f_*Enterobacteriaceae* (11.32%), *Burkholderia*_sp._Tr-862 (7.09%)	21425.78
Pyruvate decarboxylase	4.1.1.1	*Saccharomyces cerevisiae* (63.76%), *Rhizopus delemar* (36.24%)	1477.27	*Saccharomyces cerevisiae* (68.14%), *Monascus purpureus* (22.91%), *Aspergillus piperis* (5.86%)	674.79
Alcohol dehydrogenase	1.1.1.1	*Pediococcus pentosaceus* (39.97%), unclassified_g_*Pantoea* (10.75%), *Weissella cibaria* (9.82%), *Kosakonia cowanii* (3.58%), *Kuraishia capsulata* (2.82%), *Tortispora caseinolytica* (2.6%), *Enterobacter asburiae* (2.5%)	62550.36	*Weissella cibaria* (28.13%), *Pediococcus pentosaceus* (23.51%), *Burkholderia gladioli* (16.51%), *Weissella*_sp._DD23 (11.68%)	141995.42
Alcohol dehydrogenase (NADP+)	1.1.1.2	*Mixta calida* (27.58%), *Pantoea dispersa* (18.55%), unclassified_f_*Enterobacteriaceae* (13.77%), *Ascoidea rubescens* (7.82%), unclassified_g_Enterobacter (6.94%), *Cronobacter malonaticus* (4.6%)	19526.13	unclassified_g_*Burkholderia* (38.2%), *Mixta calida* (19.74%), *Pantoea dispersa* (16.37%), unclassified_f_*Enterobacteriaceae* (11.31%), *Klebsiella pneumoniae* (3.68%), unclassified_g_*Enterobacter* (3.27%)	20245.66
2-Isopropylmalate synthase	2.3.3.13	unclassified_g_*Pantoea* (27.43%), *Pantoea dispersa* (23.78%), *Enterobacter roggenkampii* (7.85%), unclassified_f_*Enterobacteriaceae* (6.89%), *Kosakonia cowanii* (6.06%), *Cronobacter dublinensis* (4.38%)	19021.44	unclassified_g_*Burkholderia* (55.5%), *Pantoea dispersa* (12.63%), unclassified_g_*Pantoea* (12.45%), unclassified_f_*Enterobacteriaceae* (6.03%)	31241.03

3-Isopropylmalate dehydratase	4.2.1.33	unclassified_g_*Pantoea* (37.03%), *Pantoea dispersa* (20.83%), unclassified_f_*Enterobacteriaceae* (14.62%), unclassified_g_*Enterobacter* (6.94%), *Saccharomyces arboricola* (3.93%), Cronobacter *dublinensis* (2.56%)	18204.66	*Burkholderia gladioli* (48.38%), unclassified_g_*Burkholderia* (15.04%), unclassified_g_*Pantoea* (13.97%), *Pantoea dispersa* (10.07%), *Leuconostoc citreum* (2.87%)	39682.44
3-Isopropylmalate dehydrogenase	1.1.1.85	unclassified_g_*Pantoea* (48.52%), *Kosakonia*_sp._CCTCC M2018092 (12%), *Enterobacter asburiae* (9.16%), *Saccharomycopsis fibuligera* (7.22%), unclassified_g_*Enterobacter* (4.63%), Cronobacter *malonaticus* (3.5%)	11662.66	*Burkholderia gladioli* (51.38%), unclassified_g_*Pantoea* (27.17%), *Kosakonia*_sp._CCTCC M2018092 (5.8%), *Leuconostoc citreum* (4.68%), unclassified_f_*Enterobacteriaceae* (2.03%)	15743.48
Leucine dehydrogenase	1.4.1.9	——	0.00	unclassified_g_Rhodococcus (100%)	1.62
Branched-chain amino acid aminotransferase	2.6.1.42	unclassified_o_*Enterobacterales* (18.78%), unclassified_g_*Pantoea* (14.48%), *Cronobacter sakazakii* (13.73%), *Vanderwaltozyma polyspora* (11.07%), *Rhizopus delemar* (9.5%), unclassified_d_*Bacteria* (8.74%)	13646.52	*Burkholderia gladioli* (45.62%), *Weissella cibaria* (44.97%), unclassified_d_*Bacteria* (3.18%), unclassified_g_*Pantoea* (1.43%), *Leuconostoc citreum* (1.23%)	38300.48
Aldehyde dehydrogenase	1.2.1.- (K00138)	*Pantoea dispersa* (28.29%), *Ascoidea rubescens* (23.4%), *Rhizopus delemar* (9.98%), unclassified_g_*Enterobacter* (9%), *Enterobacter bugandensis* (6.38%), *Cyberlindnera fabianii* (5.58%),	12258.19	*Burkholderia gladioli* (34.50%), unclassified_g_*Burkholderia* (29.51%), *Pantoea dispersa* (12.96%), unclassified_g_*Pantoea* (11.10%), *Pseudomonas aeruginosa* (5.59%), *Klebsiella quasipneumoniae* (2.52%)	29627.47
Aldehyde dehydrogenase (NAD+)	1.2.1.3	*Pantoea dispersa* (28.29%), *Ascoidea rubescens* (23.4%), *Rhizopus delemar* (9.98%), unclassified_g_*Enterobacter* (9%), *Enterobacter bugandensis* (6.38%), *Cyberlindnera fabianii* (5.58%)	12258.19	*Burkholderia gladioli* (52.42%), unclassified_g_*Burkholderia* (40.04%), *Pantoea dispersa* (2.65%), *Enterobacter asburiae* (1.20%), *Saccharomyces cerevisiae* (1.05%)	41921.48
NADP-dependent aldehyde dehydrogenase	1.2.1.4	*Saccharopolyspora*_sp._7K502 (100%)	10.40	——	0.00

methylglyoxal/glyoxal reductase	1.1.1.283	*Pediococcus pentosaceus* (97.82%), *Saccharomyces cerevisiae* (1.51%)	24962.85	*Saccharomyces cerevisiae* (64.13%), *Lactococcus lactis* (35.42%)	645.36
Transketolase	2.2.1.1	unclassified_g_*Pantoea* (15.37%), *Pediococcus pentosaceus* (13.61%), unclassified_g_*Enterobacter* (11.3%), *Enterobacter mori* (5.64%), *Cronobacter sakazakii* (5.08%), *Cronobacter dublinensis* (4.65%), *Lactobacillus plantarum* (4.29%), *Plautia stali symbiont* (4.09%)	35000.51	*Burkholderia gladioli* (36.54%), *Lelliottia aquatilis* (23.56%), unclassified_*g_Pantoea* (9.25%), unclassified_f_*Enterobacteriaceae* (4.66%), *Klebsiella pneumoniae* (2.56%), Plautia stali *symbiont* (2.52%), unclassified_g_*Enterobacter* (2.30%)	46169.27
3-Deoxy-7-phosphoheptulo nate synthase	2.5.1.54	*Weissella confusa* (23.99%), *Pantoea dispersa* (14.15%), *Pantoea*_sp._VS1 (12.34%), unclassified_g_*Pantoea* (12.15%), *Kosakonia cowanii* (4.41%), *Enterobacter hormaechei* (4.1%), unclassified_f_*Enterobacteriaceae* (3.41%), *Cronobacter dublinensis* (3.22%)	37350.39	*Burkholderia gladioli* (41.17%), *Weissella cibaria* (14.01%), *Pantoea dispersa* (9.56%), *Pantoea*_sp._VS1 (8.85%), unclassified_g_*Pantoea* (8.61%), *Klebsiella pneumoniae* (3.40%), *Lactococcus* lactis (2.09%)	40713.13
3-Dehydroquinate synthase	4.2.3.4	*Weissella confusa* (48.04%), *Pantoea dispersa* (25.15%), *Pachysolen tannophilus* (5.14%), *Kosakonia cowanii* (4.97%), *Franconibacter pulveris* (4.03%), unclassified_g_*Enterobacter* (2.76%), *Weissella cibaria* (2.59%), *Lactococcus lactis* (1.77%)	21039.64	*Weissella cibaria* (50.06%), unclassified_g_*Burkholderia* (26.26%), *Pantoea dispersa* (12.37%), unclassified_f_*Enterobacteriaceae* (3.03%), *Leuconostoc citreum* (1.57%)	33219.05

3-Dehydroquinate dehydratase	4.2.1.10	*Weissella confusa* (31.99%), *Pantoea agglomerans* (17.66%), *Pantoea* dispersa (12.28%), unclassified_f_*Enterobacteriaceae* (9.32%), *Pachysolen tannophilus* (7.04%), unclassified_g_*Enterobacter* (4.68%), *Cronobacter sakazakii* (2.83%), unclassified_g_*Cronobacter* (2.31%), *Weissella cibaria* (2.17%)	15359.69	*Weissella cibaria* (39.60%), unclassified_g_*Burkholderia* (15.99%), *Burkholderia gladioli* (15.61%), *Pantoea dispersa* (8.00%), *Pantoea agglomerans* (6.77%), *Klebsiella pneumoniae* (4.71%), unclassified_f_*Enterobacteriaceae* (3.15%)	28055.45
Quinate dehydrogenase	1.1.1.24	*Aspergillus oryzae* (100%)	5.27	——	0.00
Shikimate dehydrogenase	1.1.1.25	*Weissella confusa* (41.43%), *Pantoea dispersa* (20.43%), *Enterobacter roggenkampii* (6.19%), *Cronobacter dublinensis* (5%), *Pachysolen tannophilus* (4.91%), *Enterobacter asburiae* (4.1%), *Kosakonia cowanii* (2.82%), *Weissella cibaria* (2.26%), *Lactococcus lactis* (1.69%)	22036.96	*Burkholderia gladioli* (54.02%), *Weissella cibaria* (19.14%), unclassified_g_*Burkholderia* (10.83%), *Pantoea dispersa* (5.63%), *Klebsiella pneumoniae* (2.6%)	66011.16
Shikimate kinase	2.7.1.71	unclassified_g_*Pantoea* (25.94%), *Weissella confusa* (24.56%), *Pachysolen tannophilus* (7.38%), *Enterobacteriaceae bacterium* (7.3%), unclassified_f_*Enterobacteriaceae* (5.83%), unclassified_g_*Enterobacter* (4.85%), *Klebsiella pneumoniae* (4.81%), *Cronobacter dublinensis* (3.12%), *Enterobacter bugandensis* (3.02%)	14647.99	*Weissella cibaria* (41.53%), *Burkholderia gladioli* (26.93%), unclassified_g_*Pantoea* (11.92%), Klebsiella *pneumoniae* (5.94%), *Enterobacteriaceae bacterium*_JKS000233 (2.85%), Leuconostoc *citreum* (2.20%)	24335.44

3-Phosphoshikimate 1-carboxyvinyltransferase	2.5.1.19	*Weissella confusa* (47.62%), *Pantoea ananatis* (24.1%), *Pachysolen tannophilus* (4.58%), *Enterobacter cloacae* complex_sp. (2.83%), *Enterobacter bugandensis* (2.82%), *Weissella cibaria* (2.68%), *Kosakonia cowanii* (2.63%), unclassified_g_*Enterobacter* (2.37%), *Cronobacter malonaticus* (2.26%)	23616.29	*Weissella cibaria* (63.33%), *Burkholderia gladioli* (17.57%), *Pantoea ananatis* (11.14%), *Leuconostoc citreum* (1.63%), Kosakonia *cowanii* (1.44%)	39740.56
chorismate synthase	4.2.3.5	*Weissella confusa* (81.59%), unclassified_g_*Enterobacter* (5.8%), *Weissella cibaria* (5.71%), *Cronobacter dublinensis* (5.31%), unclassified_g_*Kosakonia* (5.29%), *Nadsonia fulvescens* (5.03%), unclassified_g_*Pantoea* (2.62%), *Lactococcus lactis* (2.41%), *Pantoea deleyi* (2.34%)	18220.70	*Weissella cibaria* (77.63%), unclassified_g_*Burkholderia* (10.28%), unclassified_f_*Enterobacteriaceae* (2.89%), *Burkholderia pseudomallei* (2.49%)	34795.14
Chorismate mutase	5.4.99.5	unclassified_g_*Pantoea* (25.23%), *Pantoea*_sp._GL120224-02 (20.76%), *Pantoea dispersa* (14.48%), *Kosakonia cowanii* (8.24%), unclassified_g_*Cronobacter* (6.81%), *Enterobacter cloacae* (6.05%),	20972.97	*Burkholderia gladioli* (50.24%), unclassified_g_*Pantoea* (13.45%), *Pantoea*_sp._GL120224-02 (11.78%), *Pantoea dispersa* (8.34%), *Kosakonia cowanii* (2.86%), unclassified_f_*Enterobacteriaceae* (2.76%)	28837.76

Chorismate synthase	4.2.3.5	*Weissella confusa* (81.59%), unclassified_g_*Enterobacter* (5.8%), *Weissella cibaria* (5.71%), *Cronobacter dublinensis* (5.31%), unclassified_g_*Kosakonia* (5.29%), *Nadsonia fulvescens* (5.03%), unclassified_g_*Pantoea* (2.62%), *Lactococcus lactis* (2.41%), *Pantoea deleyi* (2.34%)	18220.70	*Weissella cibaria* (77.63%), unclassified_g_*Burkholderia* (10.28%), unclassified_f_*Enterobacteriaceae* (2.89%), *Burkholderia pseudomallei* (2.49%)	34795.14
Prephenate dehydratase	4.2.1.51	*Pantoea*_sp._GL120224-02 (34.26%), *Pantoea dispersa* (26.96%), *Kosakonia cowanii* (8.22%), *Enterobacter cloacae* (5.72%), unclassified_g_*Enterobacter* (4.39%), unclassified_g_*Cronobacter* (3.64%), *Ogataea polymorpha* (3%)	12710.06	*Burkholderia gladioli* (59.83%), *Pantoea*_sp._GL120224-02 (13.26%), *Pantoea dispersa* (13.02%), unclassified_f_*Enterobacteriaceae* (3.10%), *Klebsiella pneumoniae* (3.07%)	25608.44
Aspartate aminotransferase	2.6.1.1	unclassified_g_*Pantoea* (31.48%), Type-C *symbiont* of *Plautia stali* (28.17%), unclassified_g_*Enterobacter* (11.67%), *Pantoea dispersa* (7.09%), unclassified_g_*Kosakonia* (6.48%), *Cyberlindnera fabianii* (4.31%), *Enterobacter hormaechei* (2.13%)	16149.93	*Burkholderia gladioli* (45.69%), unclassified_g_*Pantoea* (21.44%), Type-C *symbiont* of *Plautia* stali (16.00%), *Pantoea dispersa* (5.18%), unclassified_g_*Enterobacter* (3.55%), unclassified_g_*Kosakonia* (3.03%)	17729.33
Histidinol-phosphate aminotransferase	2.6.1.9	unclassified_g_*Pantoea* (41.85%), *Enterobacter bugandensis* (8.95%), *Enterobacter cloacae* (8.27%), *Cronobacter dublinensis (7.39%),* *Ascoidea rubescens* (7.15%), unclassified_g_*Enterobacter* (6.31%), *Cronobacter sakazakii* (5.89%)	8642.07	*Burkholderia gladioli* (71.25%), unclassified_g_*Pantoea* (15.54%), unclassified_f_*Enterobacteriaceae* (2.76%), *Leuconostoc citreum* (2.47%), *Leuconostoc lactis* (2.32%)	23353.61
Tyrosine aminotransferase	2.6.1.5	*Wickerhamomyces anomalus* (76.48%), *Saccharomyces cerevisiae* (14%), *Rhizopus delemar* (9.52%)	890.69	*Saccharomyces cerevisiae* (82.63%), *Monascus purpureus* (11.80%), *Aspergillus niger* (4.75%)	157.17

Aromatic-amino-acid transaminase	2.6.1.57	*Pantoea*_sp._Ap-959 (54.55%), *Kosakonia cowanii* (8.85%), *Cronobacter malonaticus* (6.13%), unclassified_g_*Enterobacter* (6.03%), *Wickerhamomyces anomalus* (6.01%)	11328.15	unclassified_g_*Burkholderia* (70.16%), *Pantoea*_sp._Ap-959 (17.60%), *Klebsiella pneumoniae* (2.80%), *Klebsiella quasipneumoniae* (2.36%), unclassified_g_*Enterobacter* (1.56%)	24863.59
Aromatic amino acid aminotransferase II	2.6.1.58	*Candida haemuloni* (35.51%), [*Candida*] *auris* (32.44%), *Ascoidea rubescens* (25.34%), *Saccharomyces cerevisiae* (6.43%), *Candida orthopsilosis* (0.28%)	1852.45	*Saccharomyces cerevisiae* (98.57%), *Candida glabrata* (1.43%)	118.61
Phenylpyruvate decarboxylase	4.1.1.-(K12732)	*Babjeviella inositovora* (84.5%), *Saccharomyces cerevisiae* (15.5%), *Candida glabrata* (0%)	780.05	*Saccharomyces cerevisiae* (100.00%)	116.97
Aryl-alcohol dehydrogenase	1.1.1.90	*Pediococcus pentosaceus* (87.42%), *Weissella confusa* (12.19%), unclassified_f_*Enterobacteriaceae* (0.25%)unclassified_g_*Lactobacillus* (0.14%), *Amycolatopsis jejuensis* (0.01%)	21747.65	unclassified_f_*Enterobacteriaceae* (99.29%)	602.99
Phenylacetaldehyde dehydrogenase	1.2.1.39	unclassified_g_*Enterobacter* (72.67%), unclassified_o_*Enterobacterales* (15.97%), *Klebsiella pneumoniae* (3.87%), *Rhizopus microsporus* (3.52%), unclassified_g_*Klebsiella* (3.36%)	1868.98	*Klebsiella pneumoniae* (40.00%), unclassified_g_*Klebsiella* (27.14%), unclassified_g_*Enterobacter* (23.40%), unclassified_o_*Enterobacterales* (9.38%)	2175.63
Aldehyde dehydrogenase (NAD (P)+)	1.2.1.5	*Ascoidea rubescens* (52.53%), *Saccharomyces cerevisiae* (31.59%), *Rhizopus delemar* (15.88%)	627.61	*Saccharomyces cerevisiae* (79.97%), *Monascus purpureus* (19.09%)	272.77
glycerol dehydrogenase	1.1.1.6	*Pantoea dispersa* (64.4%), *Cronobacter malonaticus* (13.35%)	6358.58	*Weissella cibaria* (54.65%), *Pantoea dispersa* (23.99%)	16507.45
propanediol dehydratase large subunit	4.2.1.28	*Pediococcus pentosaceus* (96.54%)	9854.55	unclassified_f_*Enterobacteriaceae* (60.15%), *Klebsiella pneumoniae* (38.31%)	2319.12
1, 3-propanediol dehydrogenase	1.1.1.202	*Pediococcus pentosaceus* (99.48%)	25202.70	*Klebsiella pneumoniae* (99.53%)	985.05

	Acetolactate synthase	2.2.1.6	unclassified_g_*Pediococcus* (29.39%), unclassified_g_*Pantoea* (21.93%), *Pediococcus pentosaceus* (9.58%), unclassified_g_*Enterobacter* (5.54%), *Kosakonia cowanii* (4.1%), *Enterobacter cloacae* (3.68%), *Weissella cibaria* (3.41%), unclassified_f_*Enterobacteriaceae* (2.89%)	87080.72	unclassified_g_*Burkholderia* (23.39%), *Weissella cibaria* (21.11%), *Burkholderia gladioli* (20.33%), unclassified_g_*Pantoea* (15.35%), unclassified_f_*Enterobacteriaceae* (3.73%), *Kosakonia cowanii* (2.44%)	87086.38
	Ketol-acid reductoisomerase (NADP+)	1.1.1.86	*Kosakonia cowanii* (26.74%), *Pantoea*_sp._ARC607 (12.28%), *Enterobacter hormaechei* (12.03%), unclassified_g_*Cronobacter* (10.89%), [*Candida*] *haemuloni* (7.75%), *Pantoea ananatis* (6.37%), *Pantoea agglomerans* (5.89%), *Klebsiella pneumoniae* (5.35%)	9156.39	unclassified_g_*Burkholderia* (65.63%), *Kosakonia cowanii* (10.82%), *Enterobacter hormaechei* (5.77%), *Pantoea*_sp._ARC607 (4.59%), *Pantoea ananatis* (3.28%)	12222.15
Valine biosynthesis	Dihydroxy-acid dehydratase	4.2.1.9	*Pantoea*_sp._R102 (28%), *Enterobacter cloacae* (18.38%), *Salmonella* enterica (7.23%), unclassified_d_*Bacteria* (4.98%), *Hanseniaspora osmophila* (4.83%), *Kwoniella mangrovensis* (4.72%), *Escherichia* coli (4.63%), *Pantoea ananatis* (3.84%)	16159.34	*Burkholderia gladioli* (51.92%), *Burkholderia*_sp._SJZ089 (23.81%), *Pantoea*_sp._R102 (9.23%), unclassified_d_*Bacteria* (4.23%), *Enterobacter cloacae* (2.80%)	34389.18
	Valine-pyruvate aminotransferase	2.6.1.66	*Enterobacter ludwigii* (37.66%), *Pantoea dispersa* (24.83%), unclassified_f_*Enterobacteriaceae* (15.96%), *Enterobacter asburiae* (6.19%), *Cronobacter malonaticus* (3.4%), *Cronobacter sakazakii* (2.79%), Enterobacter_sp._Acro-832 (1.78%), *Kosakonia cowanii* (1.48%)	19207.34	*Burkholderia gladioli* (31.91%), *Enterobacter ludwigii* (23.71%), *Pantoea dispersa* (16.96%), unclassified_f_*Enterobacteriaceae* (11.32%), *Burkholderia*_sp._Tr-862 (7.09%)	21425.78

	Branched-chain amino acid aminotransferase	2.6.1.42	unclassified_o_*Enterobacterales* (18.78%), unclassified_g_*Pantoea* (14.48%), *Cronobacter sakazakii* (13.73%), *Vanderwaltozyma polyspora* (11.07%), *Rhizopus delemar* (9.5%), unclassified_d_*Bacteria* (8.74%)	6172.38	Burkholderia *gladioli* (45.62%), *Weissella cibaria* (44.97%), unclassified_d_*Bacteria* (3.18%), unclassified_g_*Pantoea* (1.43%), *Leuconostoc citreum* (1.23%)	38300.48
	Valine dehydrogenase (NAD+)	1.4.1.23	*Rhodococcus qingshengii* (100%)	1.73	*Rhodococcus qingshengii* (100.00%)	5.00
	Acetolactate synthase	2.2.1.6	unclassified_g_*Pediococcus* (29.39%), unclassified_g_*Pantoea* (21.93%), *Pediococcus pentosaceus* (9.58%), unclassified_g_*Enterobacter* (5.54%), *Kosakonia cowanii* (4.1%), *Enterobacter cloacae* (3.68%), *Weissella cibaria* (3.41%), unclassified_f_*Enterobacteriaceae* (2.89%)	87080.72	unclassified_g_*Burkholderia* (23.39%), *Weissella cibaria* (21.11%), *Burkholderia gladioli* (20.33%), unclassified_g_*Pantoea* (15.35%), unclassified_f_*Enterobacteriaceae* (3.73%), *Kosakonia cowanii* (2.44%)	87086.38
Leucine biosynthesis	Ketol-acid reductoisomerase (NADP+)	1.1.1.86	*Kosakonia cowanii* (26.74%), *Pantoea*_sp._ARC607 (12.28%), *Enterobacter hormaechei* (12.03%), unclassified_g_*Cronobacter* (10.89%), *Candida haemuloni* (7.75%), *Pantoea ananatis* (6.37%), *Pantoea agglomerans* (5.89%), *Klebsiella pneumoniae* (5.35%)	9156.39	unclassified_g_*Burkholderia* (65.63%), *Kosakonia cowanii* (10.82%), *Enterobacter hormaechei* (5.77%), *Pantoea*_sp._ARC607 (4.59%), *Pantoea ananatis* (3.28%)	12222.15
	Dihydroxy-acid dehydratase	4.2.1.9	*Pantoea*_sp._R102 (28%), *Enterobacter cloacae* (18.38%), *Salmonella enterica* (7.23%), unclassified_d_*Bacteria* (4.98%), *Hanseniaspora osmophila* (4.83%), *Kwoniella mangrovensis* (4.72%), *Escherichia coli* (4.63%), *Pantoea ananatis* (3.84%)	16159.34	*Burkholderia gladioli* (51.92%), *Burkholderia*_sp._SJZ089 (23.81%), *Pantoea*_sp._R102 (9.23%), unclassified_d_*Bacteria* (4.23%), *Enterobacter cloacae* (2.80%)	34389.18

	2-Isopropylmalate synthase	2.3.3.13	unclassified_g_*Pantoea* (27.43%), *Pantoea dispersa* (23.78%), *Enterobacter roggenkampii* (7.85%), unclassified_f_*Enterobacteriaceae* (6.89%), *Kosakonia cowanii* (6.06%), *Cronobacter dublinensis* (4.38%)	19021.44	unclassified_g_*Burkholderia* (55.50%), *Pantoea dispersa* (12.63%), unclassified_g_*Pantoea* (12.45%), unclassified_f_*Enterobacteriaceae* (6.03%), *Leuconostoc citreum* (2.11%), *Klebsiella pneumoniae* (2.03%)	31241.03
	3-Isopropylmalate dehydratase	4.2.1.33	unclassified_g_*Pantoea* (37.03%), *Pantoea dispersa* (20.83%), unclassified_f_*Enterobacteriaceae* (14.62%), unclassified_g_*Enterobacter* (6.94%), *Saccharomyces arboricola* (3.93%), *Cronobacter dublinensis* (2.56%)	18204.66	Burkholderia *gladioli* (48.38%), unclassified_g_*Burkholderia* (15.04%), unclassified_g_*Pantoea* (13.97%), *Pantoea dispersa* (10.07%), *Leuconostoc citreum* (2.87%), unclassified_f_*Enterobacteriaceae* (2.78%)	39682.44
	3-Isopropylmalate dehydrogenase	1.1.1.85	unclassified_g_*Pantoea* (48.52%), *Kosakonia*_sp._CCTCC M2018092 (12%), *Enterobacter asburiae* (9.16%), *Saccharomycopsis fibuligera* (7.22%), unclassified_g_Enterobacter (4.63%), *Cronobacter* malonaticus (3.5%)	11662.66	*Burkholderia gladioli* (51.38%), unclassified_g_*Pantoea* (27.17%), *Kosakonia*_sp._CCTCC M2018092 (5.80%), *Leuconostoc citreum* (4.68%), unclassified_f_*Enterobacteriaceae* (2.03%)	15743.48
	Branched-chain amino acid aminotransferase	2.6.1.42	unclassified_o_*Enterobacterales* (18.78%), unclassified_g_*Pantoea* (14.48%), *Cronobacter sakazakii* (13.73%), *Vanderwaltozyma polyspora* (11.07%), *Rhizopus delemar* (9.5%), unclassified_d_*Bacteria* (8.74%)	6172.38	*Burkholderia gladioli* (45.62%), *Weissella cibaria* (44.97%), unclassified_d_*Bacteria* (3.18%), unclassified_g_*Pantoea* (1.43%), *Leuconostoc citreum* (1.23%)	38300.48
	Leucine dehydrogenase	1.4.1.9	——	0.00	unclassified_g_*Rhodococcus* (100.00%)	1.62
Phenylalanine biosynthesis	Chorismate mutase	5.4.99.5	unclassified_g_*Pantoea* (25.23%), *Pantoea*_sp._GL120224-02 (20.76%), *Pantoea dispersa* (14.48%), *Kosakonia cowanii* (8.24%), unclassified_g_*Cronobacter* (6.81%), *Enterobacter* cloacae (6.05%)	20972.97	*Burkholderia gladioli* (50.24%), unclassified_g_*Pantoea* (13.45%), *Pantoea*_sp._GL120224-02 (11.78%), *Pantoea dispersa* (8.34%), *Kosakonia cowanii* (2.86%), unclassified_f_*Enterobacteriaceae* (2.76%)	28837.76

Cyclohexadienyl dehydratase	4.2.1.91	*Pantoea dispersa* (94.77%), *Pantoea agglomerans* (2.92%), *Klebsiella pneumoniae* (1.61%), unclassified_g_*Pantoea* (0.25%)	3615.52	*Burkholderia gladioli* (60.93%), *Pantoea dispersa* (30.17%), *Klebsiella pneumoniae* (7.12%), *Pantoea ananatis* (1.07%)	11053.39
Prephenate dehydratase	4.2.1.51	*Pantoea*_sp._GL120224-02 (34.26%), *Pantoea dispersa* (26.96%), *Kosakonia cowanii* (8.22%), *Enterobacter cloacae* (5.72%), unclassified_g_Enterobacter (4.39%), unclassified_g_*Cronobacter* (3.64%), *Ogataea polymorpha* (3%)	12710.06	*Burkholderia gladioli* (59.83%), *Pantoea*_sp._GL120224-02 (13.26%), *Pantoea dispersa* (13.02%), unclassified_f_*Enterobacteriaceae* (3.10%), *Klebsiella pneumoniae* (3.07%)	25608.44
Aspartate aminotransferase	2.6.1.1	unclassified_g_*Pantoea* (31.48%), Type-C *symbiont* of *Plautia stali* (28.17%), unclassified_g_*Enterobacter* (11.67%), *Pantoea dispersa* (7.09%), unclassified_g_*Kosakonia* (6.48%), *Cyberlindnera fabianii* (4.31%), *Enterobacter hormaechei* (2.13%)	16149.93	*Burkholderia* gladioli (45.69%), unclassified_g_*Pantoea* (21.44%), Type-C *symbiont* of *Plautia* stali (16.00%), *Pantoea dispersa* (5.18%), unclassified_g_*Enterobacter* (3.55%), unclassified_g_*Kosakonia* (3.03%)	17729.33
Histidinol-phosphate aminotransferase	2.6.1.9	unclassified_g_*Pantoea* (41.85%), *Enterobacter bugandensis* (8.95%), *Enterobacter cloacae* (8.27%), *Cronobacter dublinensis* (7.39%), *Ascoidea rubescens* (7.15%), unclassified_g_*Enterobacter* (6.31%), *Cronobacter sakazakii* (5.89%)	8642.07	*Burkholderia gladioli* (71.25%), unclassified_g_*Pantoea* (15.54%), unclassified_f_*Enterobacteriaceae* (2.76%), *Leuconostoc citreum* (2.47%), *Leuconostoc lactis* (2.32%)	23353.61
Aromatic amino acid aminotransferase II	2.6.1.58	*Wickerhamomyces anomalus* (76.48%), *Saccharomyces cerevisiae* (14%), *Rhizopus delemar* (9.52%)	890.69	*Saccharomyces cerevisiae* (98.57%), [*Candida*] *glabrata* (1.43%)	118.61
Aromatic-amino-acid transaminase	2.6.1.57	*Pantoea*_sp._Ap-959 (54.55%), *Kosakonia cowanii* (8.85%), *Cronobacter malonaticus* (6.13%), unclassified_g_*Enterobacter* (6.03%), *Wickerhamomyces anomalus* (6.01%)	11328.15	unclassified_g_*Burkholderia* (70.16%), *Pantoea*_sp._Ap-959 (17.60%), *Klebsiella pneumoniae* (2.80%), *Klebsiella quasipneumoniae* (2.36%), unclassified_g_*Enterobacter* (1.56%)	24863.59

	Tyrosine aminotransferase	2.6.1.5	*Wickerhamomyces anomalus* (76.48%), *Saccharomyces cerevisiae* (14%), *Rhizopus delemar* (9.52%)	890.69	*Saccharomyces* cerevisiae (82.63%), *Monascus purpureus* (11.80%), *Aspergillus niger* (4.75%)	157.17
	Acetyl-CoA carboxylase	6.4.1.2	unclassified_g_*Pediococcus* (33.35%), *Weissella confusa* (23.58%), *Pediococcus pentosaceus* (19.74%), unclassified_g_*Pantoea* (7.36%), *Cronobacter malonaticus* (1.66%), *Weissella cibaria* (1.58%), *Cronobacter sakazakii* (1.36%)	101018.93	*Weissella cibaria* (36.78%), *Burkholderia gladioli* (14.08%), unclassified_g_*Burkholderia* (12.60%), *Weissella*_sp._DD23 (12.58%), unclassified_g_Weissella (11.02%), unclassified_g_*Pantoea* (4.00%)	128747.70
	Fatty acid synthase, bacteria type	2.3.1.- (K11533)	*Rhodococcus erythropolis* (87.39%)*Rhodococcus*_sp._KB6 (12.61%)	41.82	*Rhodococcus erythropolis* (100.00%)	22.92
Fatty acids biosynthesis	Fatty acid synthase	2.3.1.86	*Rhizopus delemar* (34.44%), *Pichia kudriavzevii* (21.78%), *Wickerhamomyces anomalus* (11.51%), *Pachysolen tannophilus* (11.27%), *Lichtheimia ramosa* (7.8%), *Kuraishia capsulata* (7.65%)	5154.01	*Monascus purpureus* (35.28%), *Lichtheimia ramosa* (20.75%), *Saccharomyces cerevisiae* (20.58%), *Monascus ruber* (8.69%), *Monascus pilosus* (8.69%), *Aspergillus oryzae* (2.71%)	994.45
	[Acyl-carrier-protein] S-malonyltransferase	2.3.1.39	*Pediococcus pentosaceus* (50.89%), *Weissella confusa* (23.31%), *Salmonella enterica* (10.17%), unclassified_g_*Enterobacter* (4.1%), *Enterobacter cloacae* (3.76%)	41510.78	*Burkholderia gladioli* (42.43%), unclassified_g_*Burkholderia* (27.31%), *Salmonella enterica* (12.63%), unclassified_f_Enterobacteriaceae (3.06%), *Enterobacter cloacae* (2.95%), *Leuconostoc lactis* (2.35%), *Leuconostoc citreum* (2.24%)	28781.71
	3-Oxoacyl-[acyl-carrier-pro tein] synthase III	2.3.1.180	*Pediococcus pentosaceus* (65.09%), *Weissella confusa* (15.67%), *Pantoea dispersa* (5.64%), *Enterobacter cloacae complex*_sp. (3.22%), *Weissella cibaria* (2.45%)	62891.89	*Weissella cibaria* (50.87%), unclassified_g_*Burkholderia* (26.58%), *Burkholderia gladioli* (8.49%), *Pantoea dispersa* (5.83%), *Leuconostoc citreum* (2.09%)	61529.92

3-Oxoacyl-[acyl-carrier-protein] synthase I	2.3.1.41	unclassified_g_*Pantoea* (26.35%), unclassified_g_*Enterobacter* (23.65%), *Pantoea dispersa* (21.77%), *Cronobacter dublinensis* (13.73%), *Kosakonia cowanii* (4.18%), unclassified_f_*Enterobacteriaceae* (3.2%)	19289.57	unclassified_g_*Pantoea* (36.52%), *Pantoea dispersa* (34.73%), unclassified_f_*Enterobacteriaceae* (9.01%), unclassified_g_*Enterobacter* (8.52%), *Kosakonia cowanii* (4.07%), *Enterobacter cloacae* (1.92%), *Enterobacter roggenkampii* (1.52%)	11410.65
3-Oxoacyl-[acyl-carrier-protein] synthase II	2.3.1.179	*Pediococcus pentosaceus* (43.29%), Type-C *symbiont* of *Plautia stali* (10.27%), unclassified_g_*Enterobacter* (9.5%), *Kosakonia cowanii* (7.64%), unclassified_g_*Pantoea* (7.61%), *Weissella cibaria* (6.98%)	41351.79	*Weissella cibaria* (43.17%), unclassified_*g_Burkholderia* (24.51%), Type-C *symbiont* of *Plautia* stali (9.01%), unclassified_g_*Pantoea* (6.98%), unclassified_g_*Enterobacter* (4.00%), *Kosakonia cowanii* (3.46%)	38063.01
3-Oxoacyl-[acyl-carrier protein] reductase	1.1.1.100	*Pediococcus pentosaceus* (38.52%), unclassified_g_*Pediococcus* (13.75%), *Weissella confusa* (11.1%), *Pantoea dispersa* (10%), unclassified_g_*Pantoea* (6.59%), *Pantoea rwandensis* (4.14%)	111918.02	*Burkholderia gladioli* (34.79%), unclassified_g_*Burkholderia* (18.47%), unclassified_g_*Weissella* (11.30%), *Weissella cibaria* (8.17%), *Pantoea dispersa* (7.46%), *Pantoea*_sp._BK028 (4.22%), unclassified_g_*Pantoea* (3.69%), *Pantoea rwandensis* (3.42%)	101887.41
Enoyl-[acyl-carrier protein] reductase I	1.3.1.10	*Pediococcus pentosaceus* (82.06%), *Enterobacter hormaechei* (13.92%), unclassified_g_*Pantoea* (2.11%), *Enterobacter*_sp._FS01 (1%), *Klebsiella pneumoniae* (0.69%), *Lactobacillus plantarum* (0.17%)	21212.73	*Enterobacter hormaechei* (43.15%), *Klebsiella pneumoniae* (19.10%), *Leuconostoc citreum* (14.86%), unclassified_g_*Pantoea* (10.22%), unclassified_g_*Lactococcus* (7.28%), *Enterobacter*_sp._FS01 (4.44%)	3100.73

	Enoyl-[acyl-carrier protein] reductase II	1.3.1.9	*Pediococcus pentosaceus* (60.61%), *Weissella confusa* (26.43%), *Enterobacter hormaechei* (8.44%), *Enterobacter asburiae* (1.69%), unclassified_g_*Pantoea* (1.28%), *Enterobacter*_sp._FS01 (0.61%)	34975.00	unclassified_g_*Burkholderia* (55.54%), *Enterobacter hormaechei* (8.74%), *Leuconostoc citreum* (7.20%), *Enterobacter asburiae* (6.39%), *Weissella confusa* (6.34%), *Klebsiella pneumoniae* (3.87%), unclassified_f_*Enterobacteriaceae* (3.32%)	15313.80
	Enoyl-[acyl-carrier protein] reductase III	1.3.1.104	*Bacillus nakamurai* (100%)	2.64	——	0.00
	Medium-chain acyl-[acyl-carrier-protein] hydrolase	3.1.2.21	unclassified_g_*Pediococcus* (66.06%), *Weissella confusa* (30.59%), unclassified_g_*Weissella* (1.96%)	28381.42	unclassified_g_*Weissella* (91.43%), *Leuconostoc citreum* (3.78%), *Leuconostoc lactis* (3.56%)	15308.88
Esters biosynthesis	carboxylesterase	3.1.1.1	unclassified_g_*Lactobacillus* (41.89%), unclassified_g_*Staphylococcus* (15.02%), unclassified_g_*Bacillus*_f_*Bacillaceae* (12.75%), unclassified_g_*Burkholderia* (12.59%), *Variovorax*_sp._SCN_67-85 (6.63%), *Staphylococcus kloosii* (4.8%)	54.91	unclassified_g_*Burkholderia* (51.96%), *Burkholderia gladioli* (47.98%)	16289.39
	triacylglycerol lipase	3.1.1.3	*Hyphopichia burtonii* (16.19%), *Enterobacter cloacae* (15.81%), *Pachysolen tannophilus* (13.9%), *Rhizopus delemar* (11.04%), *Saccharomycodes ludwigii* (9.64%), *Saccharomyces cerevisiae* (8.77%), *Komagataella pastoris* (6.96%)	5039.63	*Burkholderia gladioli* (79.81%), unclassified_o_*Enterobacterales* (8.76%), *Saccharomyces cerevisiae* (6.14%)	9129.80
	alcohol O-acetyltransferase	2.3.1.84	*Saccharomyces cerevisiae* (100%)	251.21	*Saccharomyces cerevisiae* (100%)	295.58

	alcohol dehydrogenase	1.1.1.1	*Pediococcus pentosaceus* (39.97%), unclassified_g_*Pantoea* (10.75%), *Weissella cibaria* (9.82%), *Kosakonia cowanii* (3.58%), *Kuraishia capsulata* (2.82%), *Tortispora caseinolytica* (2.6%), *Enterobacter asburiae* (2.5%)	62550.36	*Weissella cibaria* (28.13%), *Pediococcus pentosaceus* (23.51%), *Burkholderia gladioli* (16.51%), Weissella_sp._DD23 (11.68%)	141995.42
	4-coumarate--CoA ligase	6.2.1.12	*Rhizopus stolonifer* (44.78%), *Rhizopus delemar* (5.42%)	161.07	*Saccharomyces cerevisiae* (57.55%), *Monascus purpureus* (42.45%)	45.25
	salicylate hydroxylase	1.14.13.1	*Aspergillus niger* (100%)	5.27	*Monascus purpureus* (100%)	273.74
	feruloyl esterase	3.1.1.73	*Aspergillus awamori* (100%)	2.64	*Monascus purpureus* (97.65%)	68.72
	ferulic acid decarboxylase	4.1.1.102	*Saccharomyces cerevisiae* (100%)	86.13	*Saccharomyces cerevisiae* (57.55%), *Monascus purpureus* (42.45%)	152.78
	catechol O-methyltransferase	2.1.1.6	——	0.00	*Monascus purpureus* (100%)	20.00
Phenolic metabolism	anthranilate 1, 2-dioxygenase (deaminating, decarboxylating) large subunit	1.14.12.1	unclassified_g_*Burkholderia* (100%)	14.33	unclassified_g_*Burkholderia* (99.82%)	14496.41
	dihydroxycyclohexadiene carboxylate dehydrogenase	1.3.1.25	unclassified_g_*Klebsiella* (82.61%), *Enterobacteriaceae bacterium*_S05 (11.92%), unclassified_g_*Burkholderia* (5.47%)	79.77	unclassified_g_*Burkholderia* (92.46%), unclassified_g_*Klebsiella* (7.47%)	6816.59
	shikimate dehydrogenase	1.1.1.25	*Weissella confusa* (41.43%), *Pantoea dispersa* (20.43%), *Enterobacter roggenkampii* (6.19%), *Cronobacter dublinensis* (5%), *Pachysolen tannophilus* (4.91%), *Enterobacter asburiae* (4.1%), *Kosakonia cowanii* (2.82%), *Weissella cibaria* (2.26%), *Lactococcus lactis* (1.69%)	22036.96	*Burkholderia gladioli* (54.02%), *Weissella cibaria* (19.14%), unclassified_g_*Burkholderia* (10.83%), *Pantoea dispersa* (5.63%), *Klebsiella pneumoniae* (2.6%)	66011.16

aldehyde dehydrogenase (NAD (P)+)	1.2.1.5	*Ascoidea rubescens* (52.53%), *Saccharomyces cerevisiae* (31.59%), *Rhizopus delemar* (15.88%)	627.61	*Saccharomyces cerevisiae* (79.97%), *Monascus purpureus* (19.09%)	272.77
6-methylsalicylate decarboxylase	4.1.1.52	*Kosakonia cowanii* (96.99%), unclassified_g_*Kosakonia* (10.76%)	315.90	*Kosakonia cowanii* (100%)	276.97
6-methylsalicylic acid synthase	2.3.1.165	*Aspergillus niger* (100%)	2.64	——	0.00
coniferyl-aldehyde dehydrogenase	1.2.1.68	unclassified_g_*Kosakonia* (70.92%), *Rhodococcus*_sp._IC4 135 (11%), *Burkholderia gladioli* (10.85%), *Lactobacillus plantarum* (7.23%)	47.94	*Burkholderia gladioli* (98.65%)	9206.91
isochorismate pyruvate lyase	4.2.99.21	*Pantoea vagans* (40.87%), *Kosakonia cowanii* (32.73%), *Klebsiella pneumoniae* (15.49%), *Curtobacterium plantarum* (10.92%)	91.30	[*Curtobacterium*] *plantarum* (62.17%), *Kosakonia cowanii* (37.83%)	34.96
tannase	3.1.1.20	*Aspergillus wentii* (100%)	763.31	——	0.00
vanillin dehydrogenase	1.2.1.67	*Burkholderia gladioli* (72.39%), *Bacillus amyloliquefaciens* (27.61%)	9.55	*Burkholderia gladioli* (100%)	7547.68

Table S2. Microbial contribution to the enzymes for the metabolism of biogenic amines during Hongqu rice wine and Xiaoqu rice wine brewing

			XQW		HQW	
**Pathway**	**Enzyme Name**	**Enzyme No.**	**Distribution of microbes**	**EC abundance**	**Distribution of microbes**	**EC abundance**
Bioamine degradation	primary-amine oxidase	1.4.3.21	*Rhizopus delemar* (19.38%), *Enterobacter cloacae* (14.73%), *Enterobacter kobei* (14.67%), *Enterobacter asburiae* (13.32%), *Enterobacter hormaechei* (9.34%), *Pachysolen tannophilus* (9.31%), *Enterobacter ludwigii* (7.63%), unclassified_g_*Klebsiella* (4.52%), *Lichtheimia ramosa* (3.88%)	3663.80	unclassified_g_*Klebsiella* (33%), *Enterobacter asburiae* (16.48%), *Monascus purpureus* (13.12%), *Enterobacter*_sp._18A13 (11.33%), unclassified_g_*Enterobacter* (7.3%), *Enterobacter cloacae* (6.42%), *Enterobacter kobei* (6.26%)	2089.82

monoamine oxidase	1.4.3.4	*Staphylococcus gallinarum* (100%)	1.91	*Monascus purpureus* (100%)	84.58
spermidine synthase	2.5.1.16	*Pantoea*_sp._ICBG 985 (61.22%), *Kosakonia cowanii* (9.82%), *Cronobacter malonaticus* (8.25%), *Wickerhamomyces ciferrii* (7.67%), unclassified_f_*Enterobacteriaceae* (6.42%), *Enterobacter hormaechei* (3.18%)	2601.97	unclassified_g_*Burkholderia* (39.27%), *Burkholderia gladioli* (35.08%), *Pantoea*_sp._ICBG 985 (18.75%), unclassified_f_*Enterobacteriaceae* (3.09%)	21139.65
spermidine dehydrogenase	1.5.99.6	*Cronobacter dublinensis* (41.07%), *Cronobacter sakazakii* (58.93%)	8.83	*Cronobacter sakazakii* (72.89%), *Cronobacter dublinensis* (27.11%)	47.86
polyamine oxidase	1.5.3.14	*Rhizopus delemar* (82.22%), *Lichtheimia ramosa* (17.78%)	233.76	——	0.00

glutathionylspermidine amidase/synthetase	6.3.1.8	unclassified_g_*Enterobacter* (34.56%), *Enterobacter cloacae* (23.97%), *Enterobacter*_sp._WCHEn090032 (20.55%), *Salmonella enterica* (8.76%), *Enterobacter*_sp._50588862 (5.27%), unclassified_f_*Enterobacteriaceae* (2.74%)	2989.36	unclassified_f_*Enterobacteriaceae* (32.61%), *Enterobacter cloacae* (22.99%), unclassified_g_*Enterobacter* (13.45%), *Enterobacter*_sp._WCHEn090032 (13.14%), *Klebsiella pneumoniae* (8.32%)	1794.21
gamma-glutamylputrescine synthase	6.3.1.11	*Pantoea dispersa* (36.17%), *Enterobacter roggenkampii* (13.83%), *Cronobacter dublinensis* (11.59%), *Enterobacter cloacae* (7.99%), *Enterobacter cloacae* (6.46%), *Cronobacter malonaticus* (5.62%), unclassified_g_*Enterobacter* (4.37%)	12865.38	*Pantoea dispersa* (39.09%), *Leclercia adecarboxylata* (28.19%), unclassified_f_*Enterobacteriaceae* (8.74%), *Cronobacter malonaticus* (3.75%), *Enterobacter cloacae* (3.43%)	5121.06

diamine N-acetyltransferase	2.3.1.57	*Pediococcus pentosaceus* (35.58%), *Lactobacillus plantarum* (30.54%), *Weissella confusa* (25.89%), unclassified_g_*Enterobacter* (3%), unclassified_f_*Enterobacteriaceae* (1.34%), *Pantoea*_sp._Cy-640 (1.08%), *Lactococcus lactis* (1.03%)	38541.70	*Weissella cibaria* (81.36%), *Leuconostoc citreum* (4.26%), unclassified_g_*Enterobacter* (3.69%), unclassified_f_*Enterobacteriaceae* (3.46%)	8422.15
putrescine aminotransferase	2.6.1.82	*Cronobacter dublinensis* (31.58%), *Klebsiella*_cf._*planticola B*43 (20.46%), *Kosakonia cowanii* (12.93%), *Enterobacter roggenkampii* (12.18%), *Klebsiella pneumoniae* (11.94%), *Cronobacter malonaticus* (3.72%), *Citrobacter amalonaticus* (3%)	4549.62	*Klebsiella pneumoniae* (46.78%), *Klebsiella*_cf._*planticola_B*43 (21.76%), *Lactococcus lactis* (6.77%), *Kosakonia cowanii* (6.71%)	27550.18
putrescine---pyruvate transaminase	2.6.1.113	*Burkholderia gladioli* (100%)	6.92	*Enterobacter roggenkampii* (100%)	7872.57
tyrosine decarboxylase	4.1.1.25	*Lactobacillus fuchuensis* (100%)	358.26	——	0.00

Biogenic amine synthesis	aromatic-L-amino-acid/L-tryptophan decarboxylase	4.1.1.28	*Enterobacter* (100%)	5.27	*Monascus*_f_*Aspergillaceae* (57.33%), *Ascoidea* (42.67%)	38.05
	ornithine decarboxylase	4.1.1.17	*Pantoea*_sp._*ICBG* 985 (29.29%), *Enterobacter cloacae* (15.93%), *Kosakonia cowanii* (10.9%), *Enterobacter*_sp._18*A*13 (9.54%), *Cronobacter dublinensis* (8.43%), *Enterobacter hormaechei* (3.88%), *Enterobacter bugandensis* (3.81%), *Cyberlindnera jadinii* (3.25%), *Cronobacter malonaticus* (3.17%), *Enterobacter asburiae* (3.16%)	20706.58	*Pantoea*_sp._ICBG 985 (41.48%), *Enterobacter cloacae* (16.58%), *Kosakonia cowanii* (9.58%), *Klebsiella pneumoniae* (9.55%), *Enterobacter*_sp._18A13 (8.86%)	10674.32
	agmatinase	3.5.3.11	unclassified_c_*Gammaproteobacteria* (24.04%), *Ascoidea rubescens* (17.14%), *Klebsiella michiganensis* (12.21%), *Escherichia coli* (11.8%), unclassified_g_*Cronobacter* (10.7%), *Cronobacter sakazakii* (8.66%), *Enterobacter chengduensis* (7.92%), *Mixta calida* (3.15%)	4558.91	*Burkholderia gladioli* (81.88%), unclassified_c_*Gammaproteobacteria* (6.45%), *Enterobacter chengduensis* (3.74%), *Escherichia coli* (3.64%)	9700.06
	N-carbamoylputrescine amidase	3.5.1.53	*Monascus_f_Aspergillaceae* (99.51%), *Enterobacter* (0.49%)	1425.52	*Enterobacter* (90.93%), *Monascus_f_Aspergillaceae* (9.07%), *Burkholderia gladioli* (92.43%)	8935.59

polyamine oxidase	1.5.3.17	*Enterobacter* (53.3%), *Monascus_f_Aspergillaceae* (46.7%), *Pantoea dispersa* (36.17%)	780.57	*Monascus_f_Aspergillaceae* (100%)	109.5
agmatine deiminase	3.5.3.12	unclassified_g_*Pediococcus* (86.97%), *Kosakonia*_sp._CCTCC M2018092 (12.51%)	6384.76	*Burkholderia gladioli* (92.43%), *Kosakonia*_sp._CCTCC M2018092 (4.85%), *Lactococcus lactis* (2.37%)	19036.41
spermine synthase	2.5.1.22	*Pachysolen tannophilus* (86.89%), *Saccharomyces cerevisiae* (13.11%)	687.25	*Saccharomyces cerevisiae* (100%)	125.00
arginine decarboxylase	4.1.1.19	*Pantoea dispersa* (55.54%), unclassified_g_*Enterobacter* (11.57%), *Klebsiella pneumoniae* (8.91%), *Cronobacter sakazakii* (7.8%), *Cronobacter dublinensis* (5.08%), *Enterobacter hormaechei* (3.17%)	9975.26	unclassified_g_*Burkholderia* (59.21%), *Pantoea dispersa* (24%), *Klebsiella pneumoniae* (8.53%)	16224.11
spermine synthase	2.5.1.22	*Pachysolen tannophilus* (86.89%), *Saccharomyces cerevisiae* (13.11%)	687.25	*Saccharomyces cerevisiae* (100%)	125.00

aromatic-L-amino-acid/L-tryptophan decarboxylase	4.1.1.105	*Aspergillus niger* (100%)	5.27	*Saccharomyces cerevisiae* (100%)	38.05
aromatic-L-amino-acid/L-tryptophan decarboxylase	4.1.1.28	*Enterobacter* (100%)	5.27	*Monascus_f_Aspergillaceae* (57.33%), *Ascoidea* (42.67%)	38.05
lysine decarboxylase	4.1.1.18	*Pantoea dispersa* (36.75%), *Enterobacter cloacae* (36.09%), *Kosakonia cowanii* (10.46%), *Cronobacter malonaticus* (6.25%), *Klebsiella pneumoniae* (2.66%), *Enterobacter kobei* (1.82%)	10962.79	*Pantoea dispersa* (49.69%), *Klebsiella pneumoniae* (22.86%), *Enterobacter cloacae* (14.47%)	8434.07
spermidine synthase	2.5.1.16	*Pantoea*_sp._ICBG 985 (61.22%), *Kosakonia cowanii* (9.82%), *Cronobacter malonaticus* (8.25%), *Wickerhamomyces ciferrii* (7.67%), unclassified_f_*Enterobacteriaceae* (6.42%), *Enterobacter hormaechei* (3.18%)	2601.97	unclassified_g_*Burkholderia* (39.27%), *Burkholderia gladioli* (35.08%), *Pantoea*_sp._ICBG 985 (18.75%), unclassified_f_*Enterobacteriaceae* (3.09%)	21139.65
polyamine oxidase	1.5.3.16	*Rhizopus delemar* (82.22%), *Lichtheimia ramosa* (17.78%)	233.76	——	0.00

	glutathionylspermidine amidase/synthetase	3.5.1.78	unclassified_g_*Enterobacter* (34.56%), *Enterobacter cloacae* (23.97%), *Enterobacter*_sp._WCHEn090032 (20.55%), *Salmonella enterica* (8.76%), *Enterobacter*_sp._50588862 (5.27%), unclassified_f_*Enterobacteriaceae* (2.74%)	2989.36	unclassified_f_*Enterobacteriaceae* (32.61%), *Enterobacter cloacae* (22.99%), unclassified_g_*Enterobacter* (13.45%), *Enterobacter*_sp._WCHEn090032 (13.14%), *Klebsiella pneumoniae* (8.32%)	1794.21
	D-amino-acid N-acetyltransferase	2.3.1.36	*Saccharomyces cerevisiae* (100%)	200.76	*Bacillus ginsengihumi* (100%)	206.34
Amino acid degradation	alanine dehydrogenase	1.4.1.1	*Pediococcus pentosaceus* (99.74%)	16823.47	*Lactococcus garvieae* (59.6%), *Pantoea allii* (31.46%), *Burkholderia ubonensis* (8.94%)	290.69
	D-amino-acid oxidase	1.4.3.3	*Cyberlindnera fabianii* (67.57%), *Rhizopus delemar* (17.95%), *Ascoidea rubescens* (8.94%), *Rhizopus stolonifer* (5.54%)	886.28	*Monascus purpureus* (79.56%), *Aspergillus tanneri* (18.67%), *Saccharopolyspora*_sp._5K548 (1.78%)	85.65

glycine oxidase	1.4.3.19	unclassified_g_*Pantoea* (95.4%), *Pantoea*_sp._Sc1 (3.82%)	4434.97	*Burkholderia gladioli* (69.86%), unclassified_g_*Pantoea* (29.45%)	11909.7
glycine dehydrogenase	1.4.4.2	*Pantoea dispersa* (48.21%), unclassified_g_*Enterobacter* (11.52%), *Kosakonia*_sp._CCTCC M2018092 (10.57%), *Lupinus albus* (4.89%), *Enterobacter cloacae* (4.11%), *Franconibacter pulveris* (3.7%)	19222.39	*Burkholderia gladioli* (46.2%), *Pantoea dispersa* (30.84%), *Klebsiella pneumoniae* (4.4%)	121322.30
glycine C-acetyltransferase	2.3.1.29	unclassified_g_*Pantoea* (61.48%), unclassified_g_*Enterobacter* (11.66%), *Kosakonia pseudosacchari* (11.07%), *Enterobacter bugandensis* (6.38%), *Enterobacter roggenkampii* (5.28%), *Pantoea agglomerans* (2.03%)	9838.88	*Burkholderia gladioli* (59.74%), unclassified_g_*Pantoea* (28.37%), *Kosakonia pseudosacchari* (3.16%)	17015.83
glycine C-acetyltransferase	2.3.1.37	unclassified_g_*Pantoea* (61.48%), unclassified_g_*Enterobacter* (11.64%), *Kosakonia cowanii* (11.07%), *Enterobacter bugandensis* (6.38%), *Enterobacter roggenkampii* (5.28%), *Pantoea agglomerans* (2.03%)	9838.88	*Burkholderia gladioli* (59.74%), unclassified_g_*Pantoea* (28.37%), *Kosakonia cowanii* (3.16%)	17015.83

D-amino-acid oxidase	1.4.3.3	*Cyberlindnera fabianii* (67.57%), *Rhizopus delemar* (17.95%), *Ascoidea rubescens* (8.94%), *Rhizopus stolonifer* (5.54%)	886.28	*Monascus purpureus* (79.56%), *Aspergillus tanneri* (18.67%), *Saccharopolyspora*_sp._5K548 (1.78%)	85.65
glycine amidinotransferase	2.1.4.1	*Pantoea*_sp._ICBG 985 (100%)	132.55	——	0.00
arginine N-succinyltransferase	2.3.1.109	*Pantoea dispersa* (61.62%), unclassified_f_*Enterobacteriaceae* (14.41%), *Cronobacter malonaticus* (9.13%), *Enterobacter huaxiensis* (5.71%), unclassified_g_*Enterobacter* (4.82%), unclassified_g_*Klebsiella* (1.37%)	6512.18	*Burkholderia gladioli* (37.7%), unclassified_g_*Burkholderia* (35.81%), *Pantoea dispersa* (15.21%)	21547.89
nitric-oxide synthase, bacterial	1.14.14.47	unclassified_g_*Staphylococcus* (61.23%), *Bacillus amyloliquefaciens* (23.39%), *Saccharopolyspora shandongensis* (15.37%)	11.27	*Bacillus ginsengihumi* (100%)	3.30
saccharopine dehydrogenase (NAD+, L-lysine forming)	1.5.1.7	*Suhomyces tanzawaensis* (72.72%), *Rhizopus delemar* (18.35%), *Saccharomyces cerevisiae* (8.32%)	935.62	*Saccharomyces cerevisiae* (97.39%), *Monascus purpureus* (2.61%)	129.08
lysine 2-monooxygenase	1.13.12.2	*Amycolatopsis anabasis* (100%)	3.47	——	0.00

tyrosinase	1.14.18.1	——	0.00	*Aspergillus niger* (69.89%), *Monascus purpureus* (30.11%)	20.31
D-amino-acid oxidase	1.4.3.3	*Cyberlindnera fabianii* (67.57%), *Rhizopus delemar* (17.95%), *Ascoidea rubescens* (8.94%), *Rhizopus stolonifer* (5.54%)	886.28	*Monascus purpureus* (79.56%), *Aspergillus tanneri* (18.67%), *Saccharopolyspora_* sp._5K548 (1.78%)	85.65
ornithine--oxo-acid transaminase	2.6.1.13	*Cyberlindnera jadinii* (62.56%), *Rhizopus delemar* (21.58%), *Saccharomyces cerevisiae* (13.28%)	1134.43	*Saccharomyces cerevisiae* (82.51%), *Monascus purpureus* (16.67%)	197.50
histidine ammonia-lyase	4.3.1.3	*Pantoea sp.* (35.25%), *Pantoea dispersa* (28.64%), unclassified_g_*Enterobacter* (23.59%), *Klebsiella_*cf.*_planticola B*43 (5.66%)	14841.95	unclassified_g_*Burkholderia* (30.64%), *Burkholderia gladioli* (27.26%), *Pantoea sp.* (16.31%), *Pantoea dispersa* (14.54%)	27222.84
glutamate N-acetyltransferase / amino-acid N-acetyltransferase	2.3.1.1	unclassified_g_*Pantoea* (20.38%), unclassified_g_*Enterobacter* (15.55%), *Cronobacter malonaticus* (10.71%), *Lichtheimia ramosa* (9.55%), *Weissella cibaria* (6.7%), *Leuconostoc holzapfelii* (5.7%)	8791.30	*Burkholderia gladioli* (44.58%), *Weissella cibaria* (43.98%)	38619.68

carbamoyl-phosphate synthase small subunit	6.3.5.5	*Pediococcus pentosaceus* (44.96%), *Weissella confusa* (22.03%), *Ascoidea rubescens* (14.74%), unclassified_g_*Pantoea* (2.89%), *Kosakonia cowanii* (2.03%)	162641.55	*Weissella cibaria* (64.37%), unclassified_g_*Weissella* (11.74%), unclassified_g_*Burkholderia* (10.46%)	135275.24
glutamate 5-kinase	2.7.2.11	unclassified_g_*Pantoea* (39.63%), *Enterobacter roggenkampii* (18.73%), unclassified_f_*Enterobacteriaceae* (10.39%), *Cronobacter sakazakii* (10.38%), *Ascoidea rubescens* (6.47%), *Lactococcus lactis* (3.08%)	10949.68	unclassified_g_*Burkholderia* (47.92%), unclassified_g_*Pantoea* (25.61%), unclassified_f_*Enterobacteriaceae* (4.37%), *Klebsiella quasipneumoniae* (4.11%)	15192.74
aromatic amino acid aminotransferase I / 2-aminoadipate transaminase	2.6.1.27	——	0.00	*Saccharomyces cerevisiae* (82.63%), *Monascus purpureus* (11.8%), *Aspergillus niger* (4.75%)	157.17
tryptophanase	4.1.99.1	*Cronobacter dublinensis* (99.38%)	1326.21	*Pantoea ananatis* (94.3%), *Cronobacter dublinensis* (4.56%)	148.21

Amino acid synthesis	aminopeptidase N	3.4.11.2	*Pediococcus pentosaceus* (63.37%), *Pantoea dispersa* (14.06%), unclassified_g_*Enterobacter* (4.42%), *Kosakonia cowanii* (4%), *Weissella confusa* (3.3%)	38172.07	*Weissella cibaria* (42.37%), *Burkholderia gladioli* (17.48%), unclassified_g_*Burkholderia* (13.56%), *Weissella confusa* (10.36%), *Pantoea dispersa* (8.37%)	54326.38
	Aspartate aminotransferase	2.6.1.1	unclassified_g_*Pantoea* (31.48%), Type-C *symbiont* of *Plautia stali* (28.17%), unclassified_g_*Enterobacter* (11.67%), *Pantoea dispersa* (7.09%), unclassified_g_*Kosakonia* (6.48%), *Cyberlindnera fabianii* (4.31%), *Enterobacter hormaechei* (2.13%)	16149.93	*Burkholderia gladioli* (45.69%), unclassified_g_*Pantoea* (21.44%), Type-C *symbiont* of *Plautia stali* (16.00%), *Pantoea dispersa* (5.18%), unclassified_g_*Enterobacter* (3.55%), unclassified_g_*Kosakonia* (3.03%)	17729.33

Histidinol-phosphate aminotransferase	2.6.1.9	unclassified_g_*Pantoea* (41.85%), *Enterobacter bugandensis* (8.95%), *Enterobacter cloacae* (8.27%), *Cronobacter dublinensis* (7.39%), *Ascoidea rubescens* (7.15%), unclassified_g_*Enterobacter* (6.31%), *Cronobacter sakazakii* (5.89%)	8642.07	*Burkholderia gladioli* (71.25%), unclassified_g_*Pantoea* (15.54%), unclassified_f_*Enterobacteriaceae* (2.76%), *Leuconostoc citreum* (2.47%), *Leuconostoc lactis* (2.32%)	23353.61
Tyrosine aminotransferase	2.6.1.5	*Wickerhamomyces anomalus* (76.48%), *Saccharomyces cerevisiae* (14%), *Rhizopus delemar* (9.52%)	890.69	*Saccharomyces cerevisiae* (82.63%), *Monascus purpureus* (11.80%), *Aspergillus niger* (4.75%)	157.17
aromatic amino acid aminotransferase II	2.6.1.28	*Ascoidea rubescens* (60.84%), *Candida auris* (32.44%), *Saccharomyces cerevisiae* (6.43%)	1852.45	*Saccharomyces cerevisiae* (98.57%), *[Candida] glabrata* (1.43%)	118.61
chorismate mutase / prephenate dehydratase	4.2.1.51	*Pantoea*_sp._GL120224-02 (34.26%), *Pantoea dispersa* (26.96%), *Kosakonia cowanii* (8.22%), *Enterobacter cloacae* (5.72%), unclassified_g_*Enterobacter* (4.39%)	12710.06	*Burkholderia gladioli* (59.83%), *Pantoea*_sp._GL120224-02 (13.26%), *Pantoea dispersa* (13.02%)	25608.44

chorismate mutase / prephenate dehydratase	5.4.99.5	unclassified_g_*Pantoea* (25.23%), *Pantoea*_sp._GL120224-02 (20.76%), *Pantoea dispersa* (14.48%), *Kosakonia cowanii* (8.24%), unclassified_g_*Cronobacter* (6.81%), *Enterobacter cloacae* (6.05%), *Enterobacter kobei* (5.35%)	20972.97	*Burkholderia gladioli* (50.24%), unclassified_g_*Pantoea* (13.45%), *Pantoea*_sp._GL120224-02 (11.78%), *Pantoea dispersa* (8.34%)	28837.76
cyclohexadienyl dehydratase	4.2.1.91	*Pantoea dispersa* (94.77%), *Pantoea agglomerans* (2.92%), *Klebsiella pneumoniae* (1.61%)	3615.52	*Burkholderia gladioli* (60.93%), *Pantoea dispersa* (30.17%), *Klebsiella pneumoniae* (7.12%)	11053.39
phenylalanine ammonia-lyase	4.3.1.24	*Aspergillus phoenicis* (100%)	2.64	——	0.00
chorismate mutase / prephenate dehydratase	5.4.99.5	unclassified_g_*Pantoea* (25.23%), *Pantoea*_sp._GL120224-02 (20.76%), *Pantoea dispersa* (14.48%), *Kosakonia cowanii* (8.24%), unclassified_g_*Cronobacter* (6.81%), *Enterobacter cloacae* (6.05%)	20972.97	*Burkholderia gladioli* (50.24%), unclassified_g_*Pantoea* (13.45%), *Pantoea*_sp._GL120224-02 (11.78%), *Pantoea dispersa* (8.34%)	28837.76

saccharopine dehydrogenase (NAD+, L-lysine forming)	1.5.1.7	*Suhomyces tanzawaensis* (72.72%), *Rhizopus delemar* (18.35%), *Saccharomyces cerevisiae* (8.32%)	935.62	*Saccharomyces cerevisiae* (97.39%), *Monascus purpureus* (2.61%)	129.08
diaminopimelate decarboxylase	4.1.1.20	*Pediococcus pentosaceus* (48.34%), *Weissella confusa* (26.58%), unclassified_g_*Pantoea* (11.45%), *Enterobacter cloacae* (3.87%)	44971.15	*Weissella cibaria* (41.93%), *Burkholderia gladioli* (30.52%), unclassified_g_*Pantoea* (14.38%)	30099.56
aromatic-amino-acid transaminase	2.6.1.57	*Pantoea*_sp._Ap-959 (54.55%), *Kosakonia cowanii* (8.85%), *Cronobacter malonaticus* (6.13%), unclassified_g_*Enterobacter* (6.03%), *Wickerhamomyces anomalus* (6.01%)	11328.15	unclassified_g_*Burkholderia* (70.16%), *Pantoea*_sp._Ap-959 (17.6%), *Klebsiella pneumoniae* (2.8%)	24863.59
saccharopine dehydrogenase (NAD+, L-lysine forming)	1.5.1.7	*Suhomyces tanzawaensis* (72.72%), *Rhizopus delemar* (18.35%), *Saccharomyces cerevisiae* (8.32%)	935.62	*Saccharomyces cerevisiae* (97.39%), *Monascus purpureus* (2.61%)	129.08

diaminopimelate decarboxylase	4.1.1.20	*Pediococcus pentosaceus* (48.34%),<br>*Weissella confusa* (26.58%),<br>unclassified_g_*Pantoea* (11.45%),<br>*Enterobacter cloacae* (3.87%)	44971.15	*Weissella cibaria* (41.93%),<br>*Burkholderia gladioli* (30.52%),<br>unclassified_g_*Pantoea* (14.38%)	30099.56
prephenate dehydrogenase (NADP+)	1.3.1.13	*Candida tropicalis* (52.04%),<br>*Diutina rugosa* (21.9%),<br>*Pichia kudriavzevii* (10.21%),<br>*Rhizopus delemar* (6.17%),<br>*Saccharomyces cerevisiae* (5.13%),<br>*Lichtheimia ramosa* (4.57%)	1285.55	*Saccharomyces cerevisiae* (83.33%),<br>*Monascus purpureus* (16.67%)	130.48
cyclohexadieny/prephenate dehydrogenase	1.3.1.43	*Klebsiella pneumoniae* (54.61%),<br>*Acinetobacter albensis* (45.39%)	547.10	*Klebsiella pneumoniae* (100%)	13.04
phenylalanine-4-hydroxylase [EC:1.14.16.1]	1.14.16.1	*Rhizopus delemar* (97.97%),<br>*Burkholderia gladioli* (2.03%)	349.24	*Burkholderia gladioli* (100%)	7556.32
alpha-aminoadipic semialdehyde synthase	1.5.1.8	*Rhizopus delemar* (72.46%),<br>*Rhizopus microsporus* (27.54%)	54.17	——	0.00

Aspartate aminotransferase	2.6.1.1	unclassified_g_*Pantoea* (31.48%), Type-C *symbiont* of *Plautia stali* (28.17%), unclassified_g_*Enterobacter* (11.67%), *Pantoea dispersa* (7.09%), unclassified_g_*Kosakonia* (6.48%), *Cyberlindnera fabianii* (4.31%), *Enterobacter hormaechei* (2.13%)	16149.93	*Burkholderia gladioli* (45.69%), unclassified_g_*Pantoea* (21.44%), Type-C *symbiont* of *Plautia stali* (16.00%), *Pantoea dispersa* (5.18%), unclassified_g_*Enterobacter* (3.55%), unclassified_g_*Kosakonia* (3.03%)	17729.33
Histidinol-phosphate aminotransferase	2.6.1.9	unclassified_g_*Pantoea* (41.85%), *Enterobacter bugandensis* (8.95%), *Enterobacter cloacae* (8.27%), *Cronobacter dublinensis* (7.39%), *Ascoidea rubescens* (7.15%), unclassified_g_*Enterobacter* (6.31%), *Cronobacter sakazakii* (5.89%)	8642.07	*Burkholderia gladioli* (71.25%), unclassified_g_*Pantoea* (15.54%), unclassified_f_*Enterobacteriaceae* (2.76%), *Leuconostoc citreum* (2.47%), *Leuconostoc lactis* (2.32%)	23353.61
Tyrosine aminotransferase	2.6.1.5	*Wickerhamomyces anomalus* (76.48%), *Saccharomyces cerevisiae* (14%), *Rhizopus delemar* (9.52%)	890.69	*Saccharomyces cerevisiae* (82.63%), *Monascus purpureus* (11.80%), *Aspergillus niger* (4.75%)	157.17

acetylornithine deacetylase	3.5.1.16	unclassified_g_*Pantoea* (37.78%), *Pantoea dispersa* (24.93%), *Leclercia adecarboxylata* (6.94%), unclassified_f_*Enterobacteriaceae* (4.16%)	20722.09	*Burkholderia gladioli* (40.73%), unclassified_g_*Burkholderia* (18.75%), unclassified_g_*Pantoea* (16.51%), *Pantoea dispersa* (12.9%), *Klebsiella pneumoniae* (3.22%)	40955.19
glutamate N-acetyltransferase / amino-acid N-acetyltransferase	2.3.1.35	*Lichtheimia ramosa* (35.73%), *Weissella cibaria* (25.14%), *Leuconostoc holzapfelii* (21.4%), *Rhizopus delemar* (8.92%), *Saccharomyces cerevisiae* (3.68%)	2341.83	*Weissella cibaria* (60.91%), *Burkholderia gladioli* (32.81%), *Leuconostoc citreum* (2.53%)	27888.90
tryptophan synthase beta chain	4.2.1.20	unclassified_g_*Pantoea* (40.69%), unclassified_g_*Enterobacter* (13.36%), *Enterobacter ludwigii* (10.66%), *Scheffersomyces stipitis* (6.94%), *Leuconostoc lactis* (6.84%)	13438.57	unclassified_g_*Burkholderia* (37.15%), *Burkholderia gladioli* (31.11%), unclassified_g_*Pantoea* (18.41%)	22765.57

histidinol dehydrogenase	1.1.1.23	unclassified_g_*Pantoea* (41.86%), *Enterobacter asburiae* (13.26%), *Rhizopus delemar* (10.73%), *Kosakonia*_sp._CCTCC_M2018092 (7.26%), *Cronobacter sakazakii* (5.95%), *Enterobacter cloacae* (4.92%), *Enterobacter bugandensis* (4.91%)	9436.38	*Burkholderia gladioli* (55.67%), unclassified_g_*Pantoea* (25.64%), *Klebsiella pneumoniae* (4.41%)	15783.70
cytosolic nonspecific dipeptidase	3.4.13.18	*Rhizopus microsporus* (100%)	9.64	——	0.00
1-pyrroline-5-carboxylate dehydrogenase	1.2.1.88	*Pantoea dispersa* (43.26%), *Klebsiella pneumoniae* (9.04%), *Enterobacter bugandensis* (7.47%), unclassified_g_*Enterobacter* (7%), *Enterobacter*_sp._*BWH*52 (6.6%), *Ascoidea rubescens* (5.46%), *Enterobacter*_sp._N18-03635 (4.22%)	13482.70	*Burkholderia gladioli* (52.24%), *Pantoea dispersa* (23.42%), *Klebsiella pneumoniae* (7.91%), unclassified_g_*Enterobacter* (7.49%)	21362.82
glutamate dehydrogenase	1.4.1.2	*Candida albicans* (56.1%), *Rhizopus microsporus* (13.7%), *Lichtheimia ramosa* (11.02%), *Saccharomyces cerevisiae* (9.91%), *Rhizopus delemar* (6.26%)	1628.92	*Saccharomyces cerevisiae* (62.98%), *Monascus purpureus* (32.8%), *Rhodococcus erythropolis* (2.11%)	240.55

glutamate dehydrogenase (NAD(P)+)	1.4.1.3	*Rhizopus delemar* (43.25%), *Klebsiella aerogenes* (22.01%), *Pantoea*_sp._*ARC*607 (19.6%), *Pantoea agglomerans* (6.08%), unclassified_g_*Pantoea* (4.06%)	335.71	*Burkholderia plantarii* (93.25%), *Klebsiella aerogenes* (5.42%)	9975.03
glutamate dehydrogenase (NADP+)	1.4.1.4	*Enterobacter roggenkampii* (20.49%), *Enterobacter asburiae* (14.97%), *Enterobacter cloacae* (13.31%), *Hyphopichia burtonii* (13.18%), *Kosakonia cowanii* (11.88%), unclassified_g_*Enterobacter* (9.94%), *Lactococcus lactis* (6.03%), *Saccharomyces cerevisiae* (4.85%)	5255.39	unclassified_f_*Enterobacteriaceae* (28.8%), *Kosakonia cowanii* (20.28%), *Saccharomyces cerevisiae* (12.09%), *Enterobacter roggenkampii* (10.87%), *Enterobacter asburiae* (8.74%)	2194.77
glutaminase	3.5.1.2	*Pediococcus pentosaceus* (55.59%), *Weissella confusa* (23.28%), *Pantoea dispersa* (10.43%), *Kosakonia*_sp._CCTCC_M2018092 (3.03%), unclassified_g_*Enterobacter* (2.53%)	35948.49	unclassified_g_*Burkholderia* (54.56%), *Pantoea dispersa* (22.52%), *Weissella cibaria* (10.07%), *Klebsiella pneumoniae* (3.82%)	14915.00
glutamin-(asparagin-)ase [EC:3.5.1.38]	3.5.1.38	*Variovorax*_sp._SCN_67-85 (100%)	1.72	——	0.00

N-formylglutamate deformylase	3.5.1.68	unclassified_g_*Pantoea* (98.71%)	3745.14	*Burkholderia gladioli* (82.91%), unclassified_g_*Pantoea* (16.65%)	1968.56
succinylglutamate desuccinylase	3.5.1.96	*Pantoea dispersa* (53.51%), *Cronobacter dublinensis* (9.12%), *Enterobacter bugandensis* (7.82%), *Kosakonia*_sp._CCTCC M2018092 (6.67%), *Enterobacter cloacae* (5.09%), *Cronobacter sakazakii* (4.19%), *Cronobacter malonaticus* (3.23%)	6891.50	*Burkholderia gladioli* (55.58%), *Pantoea dispersa* (27.94%), *Klebsiella pneumoniae* (5.14%), unclassified_g_*Klebsiella* (2.39%)	11867.26
5-oxoprolinase (ATP-hydrolysing) subunit A	3.5.2.9	unclassified_g_*Pantoea* (34.38%), *Pantoea dispersa* (13.73%), *Pachysolen tannophilus* (11.16%), *Enterobacter cloacae* (6.98%), *Cronobacter malonaticus* (4.56%), unclassified_g_*Enterobacter* (4.29%), *Cronobacter dublinensis* (4.04%)	24511.67	*Burkholderia gladioli* (64.8%), unclassified_g_*Burkholderia* (12.15%), unclassified_g_*Pantoea* (9.88%), *Pantoea dispersa* (4.06%)	71517.25

formiminoglutamase	3.5.3.8	unclassified_g_*Enterobacter* (26.88%), *Enterobacter*_sp._18*A*13 (25.05%), *Kosakonia cowanii* (18.54%), *Enterobacter asburiae* (12.86%), *Enterobacter kobei* (7.39%), *Klebsiella pneumoniae* (2.45%), *Enterobacter cloacae* (2.14%)	3072.42	*Klebsiella pneumoniae* (36.79%), unclassified_g_*Enterobacter* (30.41%), *Kosakonia cowanii* (12.39%), *Enterobacter cloacae* (8.2%), *Enterobacter asburiae* (7.62%)	2409.35
